# Molecular Characterization of Muscle-Invasive Bladder Cancer: Key MicroRNAs, Transcription Factors, and Differentially Expressed Genes

**DOI:** 10.3390/genes17020122

**Published:** 2026-01-24

**Authors:** Venhar Gurbuz Can

**Affiliations:** Department of Medical Biology, Faculty of Medicine, Karabuk University, Karabuk 78100, Turkey; venhargurbuz@karabuk.edu.tr

**Keywords:** microRNAs, transcription factors, differentially expressed genes, bladder cancer, epigenetics

## Abstract

**Background:** The present study set out to identify key miRNAs, TFs and signaling pathways associated with bladder cancer, with a view to elucidating the networks of miRNA-TF-gene interactions that may serve as potential molecular biomarkers for disease diagnosis. **Methods:** An integrative analysis was conducted using the publicly available microarray dataset GSE130598. Expression profanalyzede analyzed from 42 muscle-invasive bladder cancer (MIBC) tissues and 42 matched adjacent normal bladder tissues. After data preprocessing and normalization, differentially expressed genes (DEGs) were identified. To identify the associated biological processes and signaling pathways, functional enrichment analyses were conducted using the Gene Ontology (GO) and Kyoto Encyclopedia of Genes and Genomes (KEGG) databases. Protein–protein interaction (PPI) network analysis was then employed to identify hub genes and key molecular interaction modules associated with bladder cancer. **Results:** MYC, TP53, SP1, E2F1, E2F3, NFKB1, and TWIST1 were identified as central transcriptional regulators, indicating their roles in controlling genes involved in cell cycle regulation, DNA damage response, and tumor progression. Several miRNA families, including miR-200, miR-17, miR-29, miR-141, and miR-548, have been identified as key post-transcriptional regulators, suggesting their involvement in oncogenic signaling and cellular differentiation. PPI network analysis revealed MAPK3, AKT1, CHEK1, CDK1, AURKA, and AURKB as hub genes associated with cell proliferation, mitotic control, and intracellular signaling. **Conclusions:** Fundamental molecular processes underlying bladder cancer pathogenesis include cell cycle control, signal transduction, and genomic stability. These findings provide insight into the molecular regulatory landscape of MIBC and highlight potential targets for diagnostic and prognostic applications.

## 1. Introduction

Bladder cancer (BC) is among the most prevalent malignancies of the urinary tract, affecting both males and females [1]. The categorization of this condition is primarily into two subtypes, based on its invasive properties: non-muscle-invasive bladder cancer (NMIBC) and muscle-invasive bladder cancer (MIBC) [2]. MIBC is an aggressive form of bladder cancer that typically develops from NMIBC or normal urothelial cells [3]. The transition from normal bladder tissue to MIBC is a multifaceted process involving a combination of genetic, epigenetic, and environmental factors [1,4]. A comprehensive understanding of the pathogenesis of MIBC is imperative for both early diagnosis and prognosis. MIBC is a condition that is often associated with a poor prognosis, with patients frequently requiring aggressive treatment modalities such as radical cystectomy, chemotherapy, and immunotherapy [5]. The identification of molecular markers and elucidation of the molecular mechanisms involved in the progression of MIBC from normal bladder tissue are crucial for the development of enhanced diagnostic tools and treatment options [6]. Moreover, the emergence of innovative therapeutic strategies, encompassing immunotherapy and targeted therapies, offers a glimmer of hope for more efficacious interventions in the future. The molecular pathogenesis of MIBC is characterized by several key genetic mutations and alterations [7]. The TP53 tumor suppressor gene is one of the most frequently mutated genes in MIBC [8]. The loss of functional TP53 has been shown to lead to several cellular changes, including cell cycle dysregulation, inhibition of apoptosis, and genomic instability. These changes, in turn, have been shown to promote tumor progression [9]. Fibroblast Growth Factor Receptor 3 (FGFR3) mutations have been identified as a prevalent genetic aberration in non-invasive bladder cancers, with the potential to also manifest in MIBC [10]. As is well established, miRNAs play a pivotal role in regulating gene expression. This is achieved through their binding to messenger RNAs (mRNAs), either inhibiting their translation or promoting their degradation [11]. miRNAs have been shown to play a crucial role in various biological processes, encompassing diverse functions such as cell proliferation, differentiation, apoptosis, and migration. The dysregulation of these genes has been implicated in the initiation and progression of tumors [12]. It is essential to note that the function of miRNAs can be classified as either oncogenes (oncomiRs) or tumor suppressors (tumor suppressor miRNAs), depending on the specific genes they target and the prevailing cellular context. For instance, miRNA-21, a oncomiR that has been extensively researched, is frequently found to be overexpressed in a variety of cancers, including BC [13]. Conversely, miRNA-34a, a known tumor suppressor miRNA, is downregulated in many cancers, including BC [14].

In this project, we undertook a comprehensive analysis of MIBC using a multifaceted approach that encompassed DEGs, TF interactions, and miRNA-target interactions. The objective of this research is to identify the key TFs and miRNAs that regulate gene expression in MIBC. This is achieved by using data from gene expression, signal pathways, interactions between miRNAs, and relationships between TFs and their target genes. This study aims to identify TFs, signaling pathways, genes, and miRNAs that regulate gene expression in MIBC using data from gene expression, miRNA interactions, and TF-target gene associations. In light of these data, we aim to elucidate the molecular mechanisms underlying bladder tumor pathogenesis, to find new prognostic and diagnostic markers, and to discover effective targeted therapies.

This study uses NanoString kinase panel data to examine miRNA–TF–DEG interactions in muscle-invasive bladder cancer through a comprehensive network approach. Our findings reveal that cell cycle and MAPK-based signaling pathways play a central role in MIBC pathogenesis, and that TFs such as MYC, TP53, E2F1, and TWIST1 are key regulators of this network. Furthermore, we demonstrate that the miR-15/16, miR-17~92, and miR-200 families shape these processes at the post-transcriptional level. This integrated analysis provides a robust basis for identifying novel diagnostic and prognostic biomarkers in bladder cancer. Unlike previous transcriptome-wide studies, this work uses a kinase-enriched NanoString panel and integrates DEGs with miRNA and TFs regulatory layers. This enables the characterization of kinase-driven regulatory networks in muscle-invasive bladder cancer to be focused.

## 2. Materials and Methods

### 2.1. Materials

Dataset: The gene expression data were downloaded from the National Center for Biotechnology Information (NCBI) and Gene Expression Omnibus (GEO) (https://www.ncbi.nlm.nih.gov/geo/, 27 January 2025) using the GSE130598 accession number. The platform of the microarray chip analyzer was GPL26612 (NanoString nCounter Human Kinase Panel). We need to emphasize that this platform only contains information on 550 kinase panel transcripts and does not encompass the entire human transcriptome. The GSE130598 NanoString dataset study comprised a total of 48 patients (disease status: MIBC—Fresh-frozen MIBC (≥pT2 stage)). The inclusion criteria for the study are as follows: Patients who underwent radical cystectomy in the application of neoadjuvant systemic therapy, and tissue samples taken with adjacent normal tissue for each sample, were screened in the nCounter System according to the manufacturer’s instructions.

**Figure 1:**
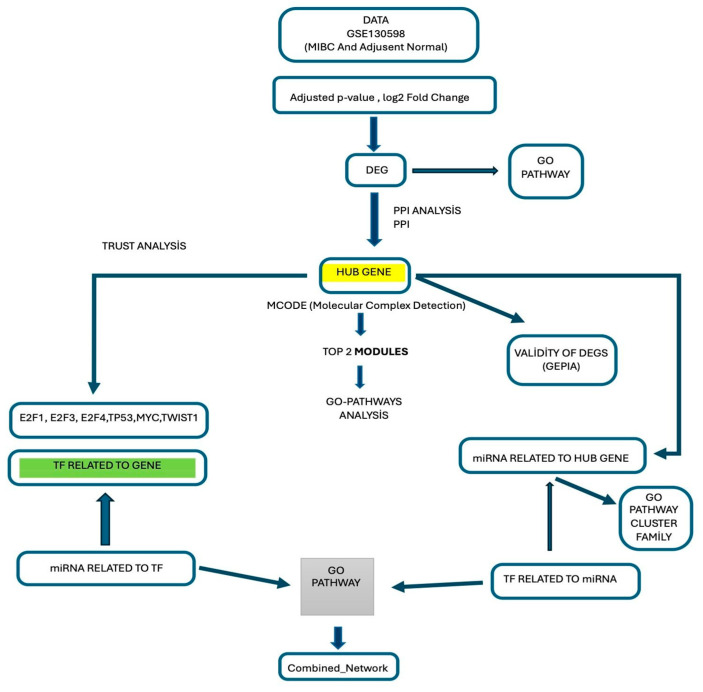
Flowchart of the bioinformatic analysis: A brief summary of our process of constructing the integrated miRNA–TF–DEG regulatory network from MIBC and adjacent normal bladder tissue GEO gene expression database. MIBC: Muscle-Invasive Bladder Cancer, DEG: Differentially Expressed Gene, PPI: Protein–Protein Interaction, MCODE: Molecular Complex Detection, GEPIA: Gene Expression Profiling Interactive Analysis, GO: Gene Ontology (Gen Ontolojisi), TF: Transcription Factor, TRRUST: Transcriptional Regulatory Relationships Unraveled by Sentence-based Text mining, miRNA: microRNA.

The gene expression data, including 42 muscle-invasive bladder cancer (MIBC) samples and 42 adjacent normal- appearing bladders, were used to analyze the differences in gene expression between these two groups. The following roadmap illustrates the structure of our study (Figure 1).

### 2.2. Methods

#### 2.2.1. Differentially Expressed Genes (DEGs)

Data Acquisition: The raw expression data from GSE130598 were obtained via the GEOquery R package (v2.66.0) (accession number: GSE130598, platform GPL26612). Enrichment analysis using GO and KEGG was performed for DEGs (using the ClusterProfiler-(V 4.19.3) and enrichR packages-(V 3.4)). HUB gene analyses were conducted utilizing the STRING database. GEPIA was utilized to validate DEGs using the TCGA (The Cancer Genome Atlas) and GTEx (Genotype-Tissue Expression) datasets. The miRDB database was used to analyze miRNAs associated with DEGs. To determine the key transcription factors (TFs) that regulate DEGs, the TRRUST v2 (Transcriptional Regulatory Relationships Unraveled by Sentence-based Text Mining) database was utilized. Only samples from platform GPL26612 (NanoString nCounter Human Kinase Panel) were used. Gene expression data were normalized using the standard NanoString nSolver pipeline, including positive-control normalization, background correction, and housekeeping gene normalization. Differential expression analysis was performed using the Limma-voom method, and multiple testing correction was applied using the Benjamini–Hochberg procedure. Genes with an adjusted *p*-value (FDR) < 0.05 and an absolute log2 fold change (|log2FC|) ≥ 1 (log2FC|) ≤ −1 were considered statistically significant. Batch effect evaluation was conducted using Uniform Manifold Approximation and Projection (UMAP), and no significant batch effects were detected; therefore, additional batch correction methods were not applied. Functional enrichment analyses for Gene Ontology and KEGG pathways were performed using the clusterProfiler and enrichR packages, with the NanoString Human Kinase Panel gene set used as the background. Protein–protein interaction analysis was conducted using the STRING database with a high-confidence interaction score threshold of 1.0 (>0.9), and network modules were identified using the MCODE algorithm in Cytoscape (ENRİCHR (V 3.4)). Transcription factor–target interactions were obtained from the TRRUST v2 database, while miRNA–target relationships were derived from miRDB using a target score greater than 80 and verified through miRBase. Gene expression and survival validation analyses were performed using the GEPIA platform based on TCGA and GTEx datasets. All analyses were conducted using RStudio (v2024.4.2).

This process resulted in the generation of a list of genes that exhibited significant upregulation and downregulation. These genes were subsequently utilized for downstream analysis. All DEGs were used to construct the network and perform further analysis.

#### 2.2.2. Enrichment Analysis

For the DEGs that have been identified, GO (Gene Ontology) analyses were conducted for biological process, molecular function, and cellular component, in addition to KEGG (Kyoto Encyclopedia of Genes and Genomes) pathway analyses. The analyses were conducted utilizing the ClusterProfiler and enrichR R packages (V 3.4). We must take into account that our DEG analysis data are related to the NanoString Human Kinase Panel, and we used only the 550 kinase-related genes found in the NanoString Human Kinase Panel rather than the entire human genome (approx. 20,000 genes) as a background gene set. This approach ensures that our enrichment analysis results indicate which pathways in the panel are truly differentially expressed, rather than a random occurrence. We acknowledge that the minimal FDR values are related to the analysis being performed on this limited universe of genes. This means that we only performed the FDR calculation on 550 genes.

#### 2.2.3. Protein–Protein Interaction (PPI) Analysis Using STRING

Given the significantly higher number of DEGs obtained, and to determine our focus point and ascertain the representative and therapeutic importance of these DEGs, PPI analyses were performed. These analyses were performed using R Studio (V2.22.0). STRING (Search Tool for the Retrieval of Interacting Genes/Proteins) is a comprehensive database and associated tool that integrates known and predicted protein–protein interactions. This analysis predicts novel interactions, thereby providing insights into undiscovered pathways or mechanisms. Proteins that possess a high degree of interconnectedness, or hub nodes, have frequently been identified as being of critical importance in the progression of diseases. There are different parameters in these analyses; the parameter we pay most attention to is setting a threshold of >0.9, which corresponds to the highest confidence (“confidence score”). For accurate and biologically meaningful interaction prediction, we used score 1. STRINGdb, (V2.22.0) the official R package for direct interface with the STRING database, is used for this analysis. The visualization of the network was conducted utilizing the Cytoscape software.

#### 2.2.4. Modular Network Analysis with MCODE

The identification of genetic clusters (modules) demonstrating intense interaction was performed by employing the MCODE (Molecular Complex Detection) algorithm on the PPI network. Subsequently, KEGG enrichment analysis was reapplied to these modules.

#### 2.2.5. Identification of Hub Genes

The 20 genes with the highest number of connections were identified through topological analysis (degree, betweenness, etc.) using the same PPI network. The biological and clinical significance of these genes was verified using the GEPIA (Gene Expression Profiling Interactive Analysis) platform.

#### 2.2.6. GEPIA (Gene Expression Profiling Interactive Analysis) Validation for Differentially Expressed Genes (DEG) and Survival Analysis

GEPIA is a critical step in biostatistical analyses to validate the findings of DEGs. GEPIA primarily validates our analysis results using large-scale genomic datasets, TCGA (The Cancer Genome Atlas) and GTEx (Genotype-Tissue Expression). The expression difference in selected genes between the BLCA (Bladder Urothelial Carcinoma) group (404 samples) and matched normal tissue groups (28 samples) was compared. In this analysis, strong thresholds were set for significance and biological difference: LogFC cutoff: 1 and statistical significance threshold *p*-value cutoff: 0.05. The results obtained from the Box Plot showed that the gene was expressed significantly differently in tumors compared to normal tissues.

The GEPIA (Gene Expression Profiling Interactive Analysis) platform was used to validate the potential clinical impact of DEGs on the survival of TCGA cancer patients. The Overall Survival method was selected for analysis, and survival curves were generated in months. Patients were divided into High-Expression and Low-Expression groups based on the Median Cutoff value of the gene’s expression level in the TCGA cohort. This means that patients were exactly divided into the 50% High Expression and 50% Low Expression groups. Kaplan–Meier curves were generated, statistical significance was determined using the Log-rank test, and the magnitude of the association was quantified using the Hazard Ratio (HR) and its 95% Confidence Interval (CI) obtained from the Cox Proportional Hazard Model (Cox PH Model). In the boxplot results obtained, quantitative data such as Hazard Ratio (HR) and *p*-value are also presented along with Kaplan–Meier curves.

#### 2.2.7. miRNA Related to DEG

The analysis of miRNA related to DEG was conducted using data from the miRDB, where predictions are made using a specially developed computational algorithm for the MirTarget dataset. A target score greater than 80 (>80) is used for selecting related miRNA, indicating that the predicted miRNA-target interaction has very high confidence. The miRBase database was used to verify the sequences and nomenclature of the obtained miRNAs in miRDB. miRBase database analyses are restricted to the Homo sapiens (Human) species.

To identify key TFs regulating DEGs, the TRRUST v2 (Transcriptional Regulatory Relationships Unraveled by Sentence-based Text mining) database, which compiles experimental and text mining data, was used. DEGs were queried in TRRUST, restricting them to Homo sapiens (Human) interactions. TFs deemed statistically significant were included in the network as those meeting the FDR threshold of ≤0.05 after FDR correction.

#### 2.2.8. TF Related to DEG

The analysis of TFs associated with DEGs is imperative for comprehending the regulatory mechanisms that underpin alterations in gene expression. TF-DEG regulatory interactions were obtained from databases such as TRRUST and TRANSFAC. The interaction network was visualized using the R packages igraph-(V2.2.1) and ggraph-(V2.2.2). Key TFs with high connectivity were selected for downstream functional analysis.

#### 2.2.9. Integrated Analysis of miRNA–TF–DEG Regulatory Network

In order to comprehend the intricate molecular regulatory mechanisms that underpin BC, a multi-layer regulatory network was constructed and analyzed, incorporating DEGs, TFs, and miRNAs. The final network was generated based on curated interactions from public databases and intersected with DEGs from the transcriptomic analysis.

## 3. Results

### 3.1. Differentially Expressed Genes (DEGs)

**Figure 2:**
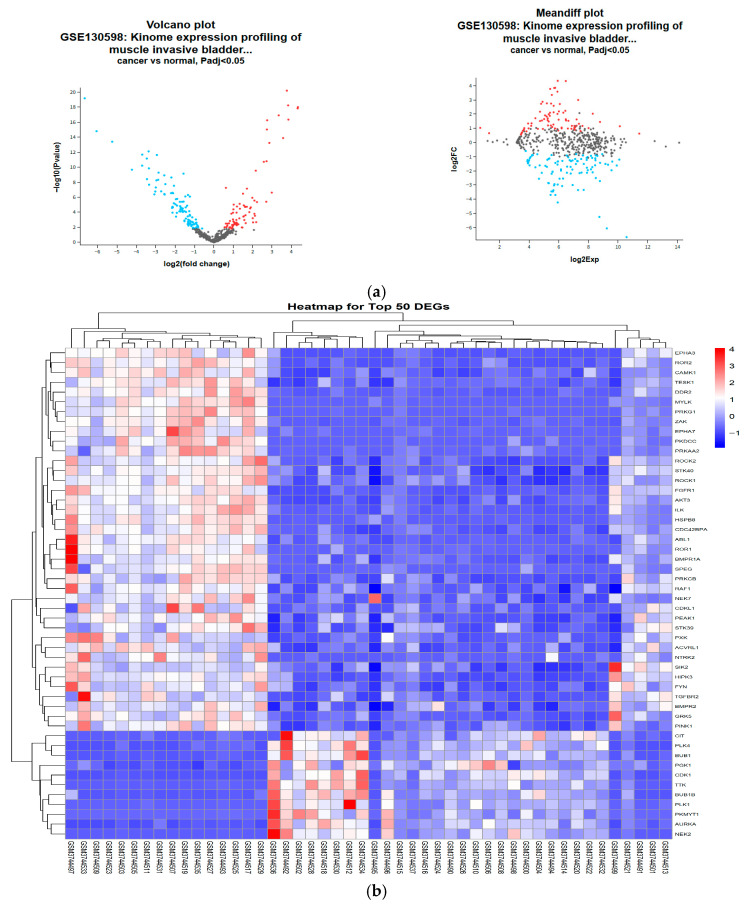
(**a**) Volcanic graph of DEGs; (**b**) Heat map of DEGs.

The GSE130598 study used the GPL26612 NanoString nCounter Human Kinase Panel. This panel examined a total of 550 genes, including kinase genes. Please note that we only used the 550 kinase-related genes found in the NanoString Human Kinase Panel and not the complete human genome (comprising approximately 20,000 genes) as the background gene set. When selecting the conditions, *p*-value (FDR) < 0.05 and |log2FC| ≥ 1, a total of 63 were found to be up-regulated, and 137 were found to be down-regulated. A total of 200 DEGs were obtained (Figure 2).

### 3.2. GO and KEGG Pathway Analyses

**Figure 3:**
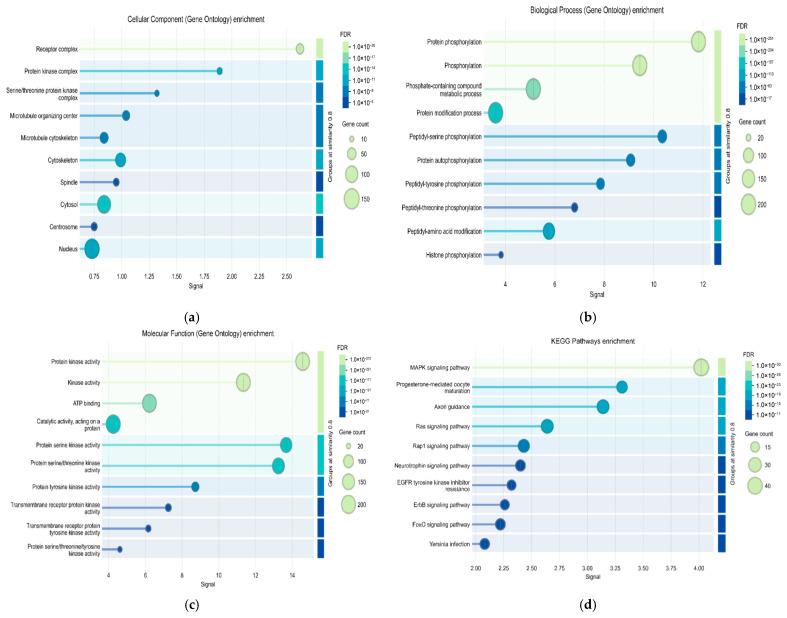
GO and KEGG analysis of the DEGs. (**a**) The Cellular Component (CC) enrichment analysis; (**b**) The Biological Process (BP) enrichment analysis; (**c**) The Molecular Function (MF) enrichment analysis; (**d**) The KEGG enrichment analysis.

In consequence of the GO analysis of the DEGs, it was determined that MAPK, an entity associated with carcinogenesis, was significantly predominant among the most enriched terms about biological processes (Figure 3, p.adj. < 0.05). The Cellular Component (CC) enrichment analysis (Figure 3a) revealed several significantly enriched cellular locations and macromolecular complexes among the DEGs. Prominently, “Receptor complex” emerged as the most highly enriched term, displaying the highest enrichment signal and an exceptionally low FDR-adjusted *p*-value (<1.0 × $10^{-20}$), indicating its robust statistical significance. This term was associated with a substantial number of DEGs. Similarly, “Protein kinase complex” and “Serine/threonine protein kinase complex” were also highly enriched, showing strong signals and significant FDR values (<1.0 × $10^{-17}$ and <1.0 × $10^{-11}$), respectively. These findings collectively highlight a significant deregulation of cellular communication and signal transduction machinery in bladder cancer. The Biological Process (BP) enrichment analysis (Figure 3b) predominantly indicated significant alterations in processes related to phosphorylation. “Protein phosphorylation” and “Phosphorylation” were among the top-enriched terms, exhibiting the highest enrichment signals and extremely low FDR-adjusted *p*-values (e.g., <1.0 × $10^{-251}$ and <1.0 × $10^{-204}$ respectively). This strong enrichment underscores that the DEGs are profoundly involved in regulating protein activity through phosphate group addition, a crucial mechanism in cell signaling and metabolism. These specific phosphorylation events suggest precise disruptions in various kinase-mediated signaling cascades within bladder cancer cells. The enrichment of “Phosphate-containing compound metabolic process” further supports the widespread metabolic reprogramming involving phosphate groups. Additionally, the terms “Protein modification process” and “Peptidyl-amino acid modification” were also significantly enriched, indicating broader changes in the post-translational modifications of proteins. The presence of “Histone phosphorylation” suggests potential epigenetic regulatory changes. The Molecular Function (MF) enrichment analysis (Figure 3c) corroborated the findings from the CC and BP analyses by highlighting altered enzymatic activities, particularly those involving kinases. “Protein kinase activity” and “Kinase activity” were the most significantly enriched terms, showing the highest enrichment signals and extremely low FDR-adjusted *p*-values (e.g., <1.0 × $10^{-272}$ and <1.0 × $10^{-221}$ respectively). This indicates that a large number of DEGs possess or contribute to the enzymatic function of phosphorylating proteins. Building on these findings, specific kinase activities—including protein serine, serine/threonine, and tyrosine kinase activities—were significantly enriched, directly aligning with the pervasive phosphorylation observed. This robust enrichment, alongside ATP binding and catalytic activity, strongly indicates altered kinase regulation and cell surface signaling in bladder cancer, consistent with cellular component findings. The KEGG enrichment analysis (Figure 3d) strongly suggests that the progression of bladder cancer involves significant activation or dysregulation of key oncogenic signaling cascades, particularly the MAPK and Ras pathways, which are central to uncontrolled cell growth and survival. Furthermore, the enrichment of developmental pathways like Axon guidance points to the activation of programs that facilitate cancer cell motility and invasion.

These findings provide a robust foundation for understanding the molecular drivers of bladder cancer and identifying potential therapeutic targets. GO analyses have revealed significant deregulation of kinase activity and phosphorylation-based processes in bladder cancer. Cellular component: Receptor and protein kinase complexes are prominent. Biological process: Protein phosphorylation, cell signaling, and post-translational modifications are predominant. Molecular function: Serine/threonine and tyrosine kinase activities are enriched. KEGG analyses have also shown that the MAPK and Ras signaling pathways, as well as the cell cycle, p53 signaling, and axon guidance pathways related to cell migration, play important roles in MIBC pathogenesis. These results are consistent with the NanoString kinase panel used in the study.

### 3.3. PPI Network and MCODE Module Analysis

**Figure 4:**
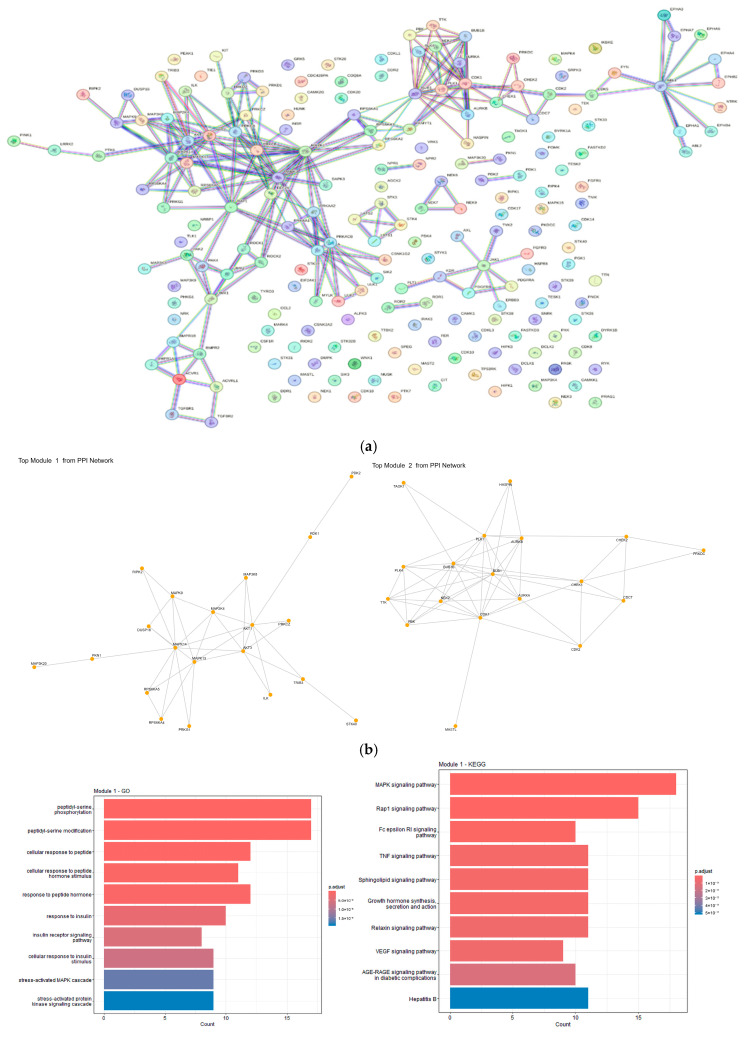

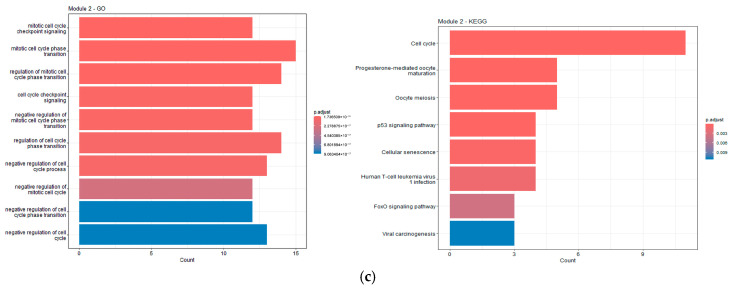
(**a**) Result View of PPI; (**b**) Module 1 and 2 NETWORK; (**c**) Modulel 1 and 2 KEGG and GO.

The protein–protein interaction (PPI) network created using the STRING database contains a total of 202 nodes and 681 connections (Figure 4). The MCODE analysis, conducted using the Cytoscape platform, has led to the identification of two modules deemed significant (hereafter referred to as Module 1 and Module 2). The first module comprises 24 nodes and 118 connections, while the second module consists of 18 nodes and 110 connections. These modules demonstrate pronounced interactions within themselves, and each has undergone KEGG analysis. Module 2 illustrates the genes involved in tumor suppression, cell cycle regulation, and the DNA damage response. In contrast, Module 1 illustrates the activation of intracellular signaling pathways (e.g., MAPK, insulin signaling pathway, and sensitivity to hormonal signals). Module 1 represents a significant cluster of interacting proteins. Its GO Biological Process enrichment highlighted a strong emphasis on phosphorylation, particularly “peptidyl-serine phosphorylation” and “peptidyl-serine modification.” The module also showed significant involvement in cellular responses to peptides and hormones, including “response to insulin” and “insulin receptor signaling pathway.” Furthermore, “stress-activated MAPK cascade” was prominently enriched, indicating its role in stress-induced signaling. KEGG pathway analysis for Module 1 reinforced the central role of the MAPK signaling pathway, the most enriched pathway. Other key enriched pathways included the “Rap1 signaling pathway,” “Fc epsilon Ri signaling pathway,” and “TNF signaling pathway,” indicating roles in cell adhesion, immune response, and inflammation. Angiogenesis was also suggested by the enrichment of “VEGF signaling pathway.” Overall, Module 1 appears to be critical for diverse signaling processes and growth regulation in bladder cancer. Module 2 formed another distinct and highly interconnected subnetwork. Its GO Biological Process enrichment strongly indicated its primary function in cell cycle control. Terms such as “mitotic cell cycle checkpoint signaling,” “mitotic cell cycle phase transition,” and “regulation of mitotic cell cycle phase transition” were highly enriched, underscoring this module’s role in governing cell division. The presence of “negative regulation of cell cycle” terms also suggests the existence of complex regulatory mechanisms within this module. KEGG pathway analysis for Module 2 conclusively confirmed its central role in the “Cell cycle” pathway, which was the most significantly enriched. Additionally, the “p53 signaling pathway” and “Cellular senescence” were enriched, highlighting the module’s involvement in critical tumor-suppressive mechanisms and cell fate. Pathways related to “Oocyte meiosis” and “Progesterone-mediated oocyte maturation” were also identified, often sharing core cell cycle machinery relevant to cancer. The enrichment of “Viral carcinogenesis” and “Human T-cell leukemia virus one infection” suggests potential links between viral oncogenesis and the observed cell cycle dysregulation in bladder cancer.

### 3.4. The Identification of Hub Genes and the GEPIA Validation

**Figure 5:**
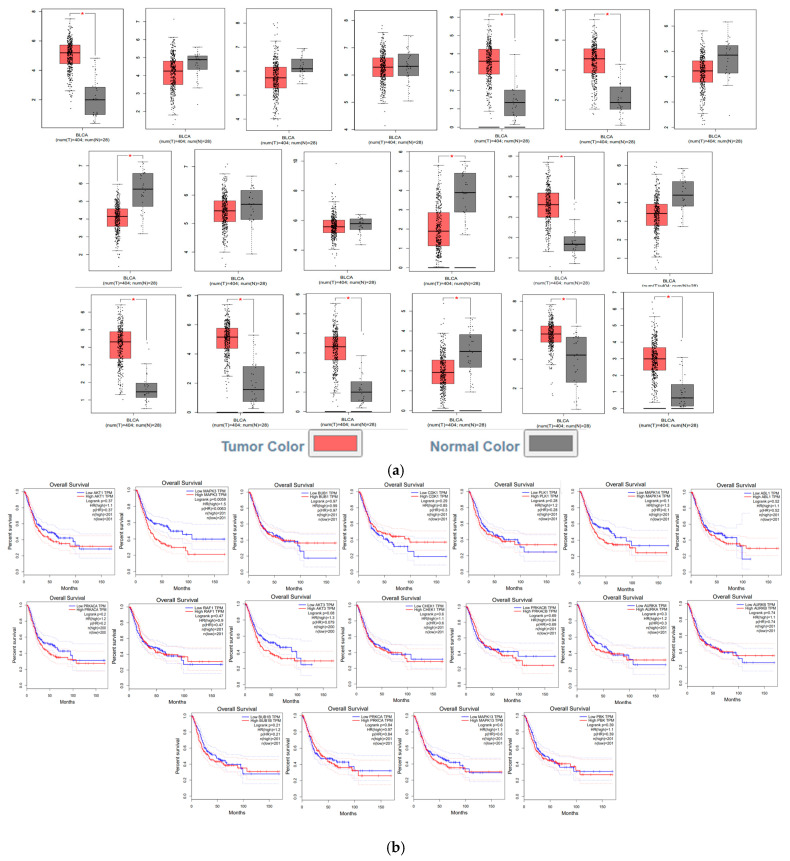
(**a**) in the order listed CDK1, MAPK1, MAPK3, AKT1, BUB1, PLK1, MAPK14, ABL1, PRKACA, RAF1, AKT3, CHEK1, PRKACB, AURKA, AURKB, BUB1B, PRKCA, MAPK13, PBK GEPIA results (Red box = Tumor (T), Gray box = Normal (N), * *p* < 0.05) and (**b**) GEPIA Survival Analysis.

A topological analysis performed via the PPI network identified 20 hub genes with the highest degree of connectivity (Table 1). These genes were found to be involved in cell processes such as cell cycle regulation, signal transduction, and proliferation. Utilizing the GEPIA platform for validation purposes, it was ascertained that, except for MAPK1 and MAPK13, these genes exhibited significant expression levels in bladder cancer tissues (*p* < 0.01). Furthermore, an association between MAPK1 and MAPK3 with overall survival was determined (Figure 5).

### 3.5. TF and miRNA Regulatory Network Associated with DEG

**Figure 6:**
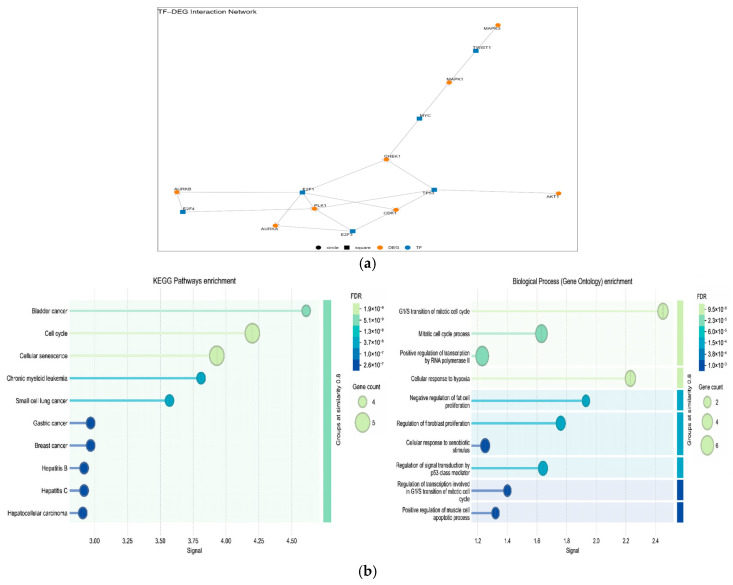
(**a**) TF-DEG interaction network; (**b**) KEGG and GO enrichment.

A total of 6 TFs and 29 miRNAs have been identified as regulators of the identified hub genes (Table 2 and Figure 6a). There is a direct relationship between the activation of the cell cycle (E2F1, E2F3), the DNA damage response (TP53, CHEK1), mitotic regulators (PLK1, AURKA, AURKB), and oncogenic signaling pathways (MYC, MAPK). The enriched KEGG pathway analysis revealed the involvement of these TFs in important pathways such as bladder cancer (illustrated below), cell cycle, and cellular senescence (Figure 6b). This section effectively moves from identifying DEGs to understanding their higher-level regulatory control. The TF-DEG network and its functional enrichment provide strong evidence that: Cell cycle regulation is a significant area of dysregulation at the transcriptional level in bladder cancer, with key TFs (like E2F1/E2F4) playing central roles. The network is directly involved in bladder cancer pathogenesis and potentially shares mechanisms with other cancers. The findings suggest specific regulatory nodes (TFs and their target DEGs) that could be critical for bladder cancer progression and potentially represent novel therapeutic targets. This study demonstrates that a multi-layered regulatory network is active in bladder cancer (particularly MIBC). Transcription factors (MYC, TP53, E2F1, E2F3, NFKB1, TWIST1) play central roles in the transcriptional regulation of genes involved in the cell cycle, DNA damage response, and tumor progression. miRNAs (particularly the miR-15/16, miR-17~92, miR-200, and miR-29 families) modulate cell proliferation, EMT, apoptosis, and signal transduction by regulating the expression of these TFs and target genes at the post-transcriptional level. KEGG analyses revealed that the MAPK, Ras, PI3K-Akt, and cell cycle pathways were predominantly affected.

### 3.6. miRNA Related to DEG

**Figure 7:**
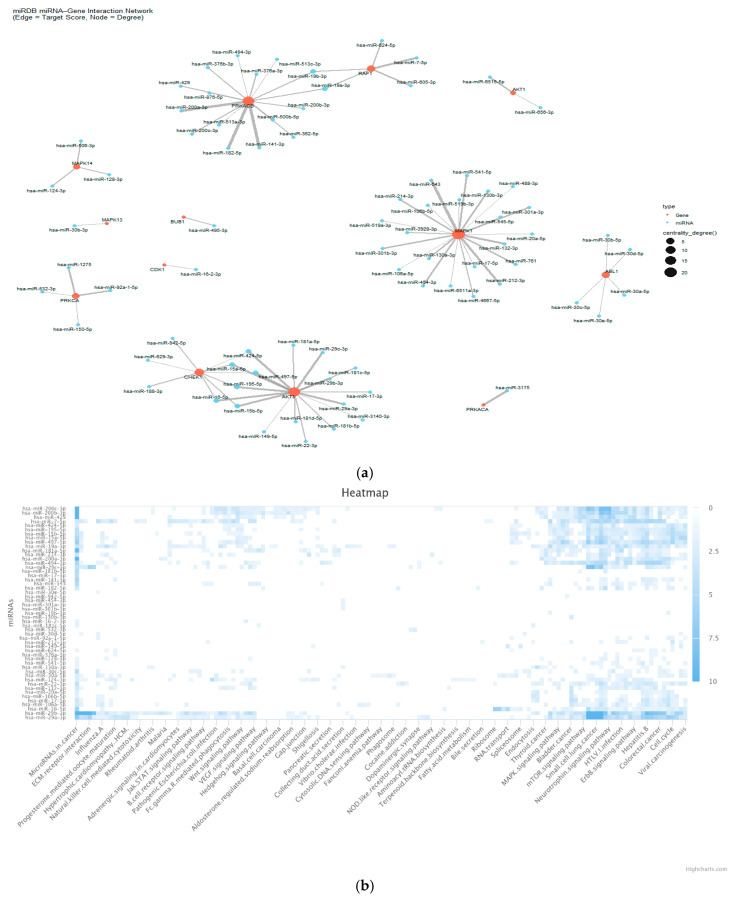
(**a**) miRNA gene interaction network; (**b**) miRNA related to DEG, KEGG analysis heatmap.

Our study yielded several significant findings. The genes identified as being of particular importance in this network are PRKAR1A, REL, AKT3, CHEK1, and MAPK1, due to their high degree of connectivity within the network. These genes are targeted by multiple miRNAs, indicating their central regulatory roles in the network (Figure 7). It is imperative to note that the following central miRNAs have been identified based on their capacity to regulate multiple target genes: hsa-miR-19b-3p, hsa-miR-19a-3p, hsa-miR-424-5p, hsa-miR-15a-5p, hsa-miR-497-5p, hsa-miR-195-5p, hsa-miR-16-5p, and hsa-miR-15b-5p. These miRNAs have been observed to regulate important signaling genes involved in cell proliferation, apoptosis, DNA damage response, and signal transduction. Based on targeting multiple genes in this specific list, miRNAs targeting both AKT3 and CHEK1 (hsa-miR-16-5p, hsa-miR-195-5p, hsa-miR-424-5p, hsa-miR-497-5p) are of interest because these genes are involved in important cancer-related pathways (PI3K/AKT and cell cycle control). Based on the heatmap obtained from the analysis of functional relationships between selected miRNA-enriched KEGG pathways, the following miRNAs were identified as having high connectivity with cancer-related pathways: hsa-miR-15a-5p, hsa-miR-16-5p, hsa-miR-195-5p, hsa-miR-497-5p, hsa-miR-19a-3p, hsa-miR-19b-3p, hsa-miR-15b-5p, and hsa-miR-424-5p. It has been demonstrated that hsa-miR-15a-5p, hsa-miR-16-5p, hsa-miR-195-5p, and hsa-miR-497-5p are strongly associated with pathways related to cell cycle regulation. This suggests that they have tumor-suppressing functions, potentially through mechanisms involving cell cycle arrest and apoptosis. Conversely, hsa-miR-19a-3p and hsa-miR-19b-3p exhibited enrichment in the MAPK, ErbB, and PI3K-Akt pathways, which are characteristic of aggressive tumor behavior. This finding indicates that these miRNAs may act as oncogenes. Based on the heatmap, hsa-miR-424-5p exhibited significant involvement in the VEGF and mTOR pathways, which are well-known key regulators of processes such as angiogenesis, cell survival, and chemotherapy resistance. This association further suggests its potential indirect role in HIF-1-related processes, given HIF-1′s integral connection to both VEGF and mTOR signaling in tumor microenvironment adaptation. Therefore, modulation of this miRNA may increase the response to chemotherapy, especially in resistant tumors. Notably, several miRNAs have been identified as effective in bladder cancer pathways, including miR-29a-3p, miR-29b-3p, miR-130a-3p, miR-7-5p, and miR-16-5p. These miRNAs are of particular significance in the context of bladder cancer biology, as they have a direct effect on disease-related pathways.

### 3.7. TF Regulating miRNAs Related to DEGs

**Figure 8:**
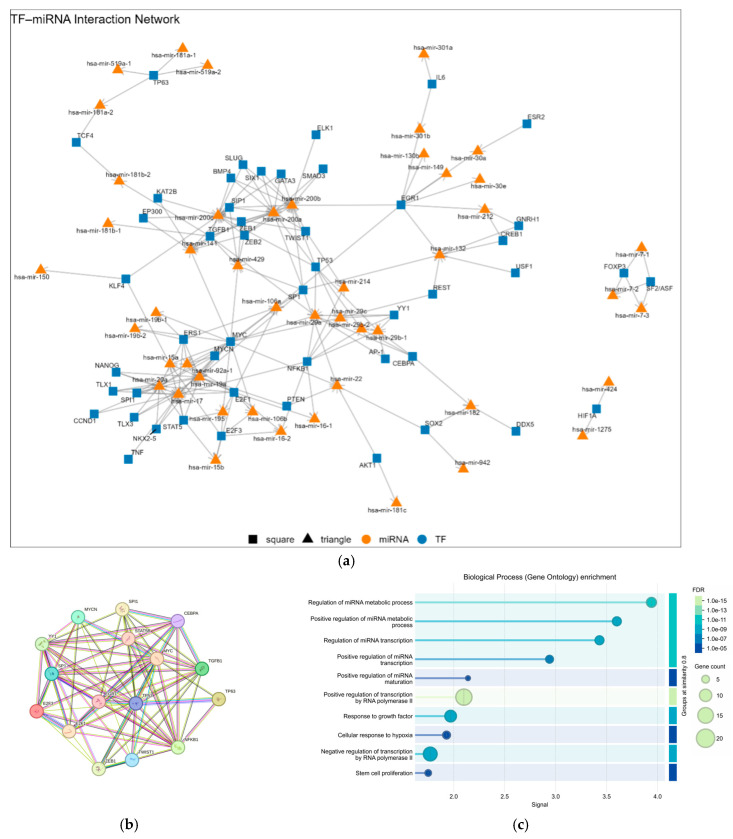
(**a**) TF miRNA interaction network; (**b**) STRİNG results (**c**) GO results.

In order to comprehend the upstream regulation of miRNAs within the network of genes, the analysis of the TFs that are predicted to regulate these particular miRNAs was conducted. Using TAM 2.0 (miRNA set enrichment analysis tool), we analyzed the results obtained from the database, considering FDR < 0.05, and identified a total of 41 different TFs regulating miRNAs associated with DEGs (Figure 8a). The key findings indicate that miRNA-29a is subject to the regulation of six distinct TFs, namely MYC, NFKB1, TGFB1, TP53, YY1, and CEBPA. These TFs have been implicated in critical cellular processes, including inflammation, proliferation, and apoptosis. The results obtained reveal a complex regulatory environment governing the expression of miRNA-29a. The members of the oncogenic miR-17~92 cluster, hsa-miR-17 and hsa-miR-20a, are each subject to regulation by six TFs; specifically, MYC, E2F1, STAT5, MYCN, NFKB1, and SPI1 for miR-17 and MYC, E2F1, STAT5, MYCN, ESR1, and SPI1 for miR-20a. The miR-200 family (including hsa-miR-200a, -200b, and -200c, as well as hsa-miR-141 and hsa-miR-429) is subject to regulation by five distinct TFs: ZEB1, TGFB1, TP53, SP1, and TWIST1. As indicated by the results obtained in the String analyses (Figure 8b), MYC and TP53 exhibited the highest binding degree. Furthermore, the GO for biological processes revealed the overrepresentation of terms related to miRNA regulation (Figure 8c).

### 3.8. miRNAs Regulating TFs Related to DEGs

**Figure 9:**
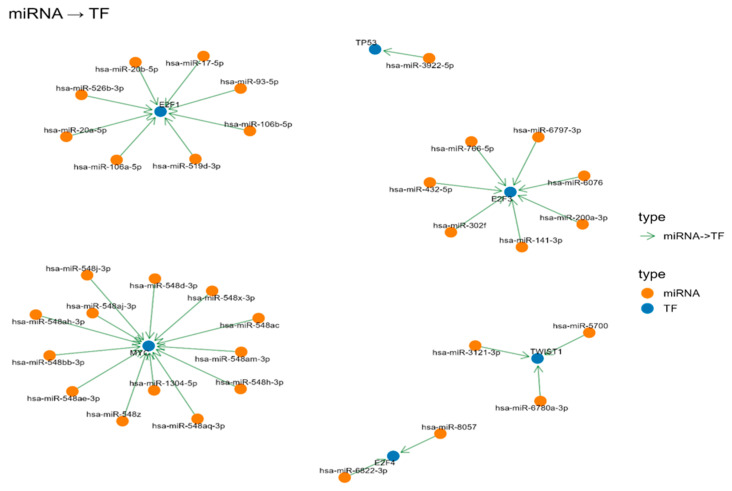
miRNA TF interaction network.

The analysis identified several miRNAs that have been predicted to target key TFs that are associated with cancer (Figure 9). In particular, a group of miRNAs, including hsa-miR-548x-3p, hsa-miR-548bb-3p, and others, have been predicted to target the oncogene MYC. Furthermore, miRNAs such as hsa-miR-17-5p and hsa-miR-106a-5p have been identified as targeting the cell cycle regulator E2F1. Furthermore, TWIST1, a TF involved in EMT, was targeted by hsa-miR-5700 and other miRNAs. Finally, the tumor suppressor TP53 was identified as a target of hsa-miR-3922-5p.

### 3.9. Integrated Analysis of miRNA–TF–DEG Regulatory Network

**Figure 10:**
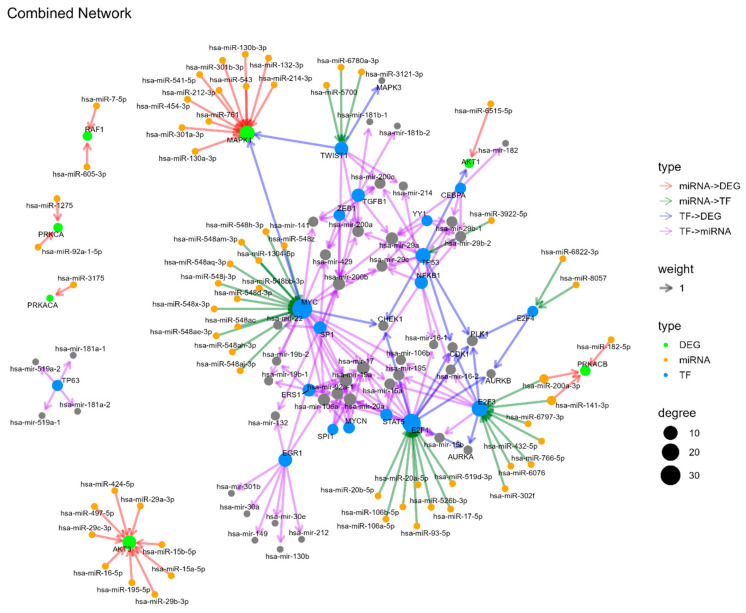
Combined network for miRNA to DEG, miRNA to TF, TF to DEG and TF to miRNA.

The integrated analysis of miRNA-TF-DEG regulatory networks (Figure 10) is of critical importance in cancer research, as it provides a comprehensive view of the multi-layered gene regulation mechanisms that drive cancer initiation, progression and metastasis. The analysis identified several high-confidence axes of miRNA-TF-DEG, including both activating and repressive interactions. The key TFs are as follows: MYC is a central TF with high connectivity to both miRNAs and DEGs. TP53, located at the center of the network with key regulatory roles, is another notable example. SP1 is a transcriptional activator that plays a role in numerous interactions between miRNAs and TFs. The genes E2F1, E2F3, NFKB1, and TWIST1 have been identified as playing significant regulatory roles in this process. The following highly connected miRNAs have been identified: hsa-miR-141-3p, the hsa-miR-17 family (hsa-miR-106b-5p, hsa-miR-106a-5p), the hsa-miR-200 family (200a/b/c, 141, 429), the hsa-miR-29 family (hsa-miR-29b, hsa-miR-29a, and hsa-miR-29c) and the hsa-miR-548 family. These miRNAs have been observed to target multiple TFs and DEGs, thereby functioning as significant regulators of downstream gene expression. The key DEGs are MAPK3, AKT1, CHEK1, CDK1, AURKA, and AURKB, which are critical DEGs affected by multiple upstream regulators and be functionally important in bladder cancer biology.

## 4. Discussion

miRNAs adopt a dualistic functionality, operating either as oncogenes or tumor suppressor genes, a distinction that arises from their capacity to influence target genes. Dysregulated miRNA expression profiles are present in various cancers, including bladder cancer. These profiles have been shown to contribute to tumor initiation, progression, metastasis and therapeutic resistance [15,16]. A significant body of research has focused on the targeting of genes expressed in bladder cancer by miRNAs, with a particular emphasis on the differences in this process compared to the targeting of bladder epithelium [17,18,19]. TFs activate or inhibit transcription via binding to domains located within the DNA helix. These domains are known as transactivation or trans-repression domains. It is noteworthy that the role of TFs extends beyond the process of DNA transcription. Recent studies have identified a growing body of evidence suggesting that TFs are playing an increasingly significant role in the development of human diseases and tumor progression [20,21,22]. For all these reasons, it is imperative to investigate the relationships between miRNAs, TFs, and target genes in bladder cancer. In this study, we conducted a comprehensive analysis to investigate the molecular mechanisms underlying bladder tumor progression, to elucidate novel prognostic and diagnostic markers, and to discover effective targeted therapies. In our study, 200 genes were identified that exhibited significant disparities between bladder cancer tissue samples and adjacent normal bladder tissue. Of these genes, 63 were identified as being overexpressed, and 137 were identified as being down-expressed. A topological analysis performed via the PPI network identified 20 hub genes with the highest degree of connectivity (Figure 7). These genes were found to be involved in cell processes such as cell cycle regulation, signal transduction, and proliferation. These modules suggest coordinated alterations in protein interaction patterns associated with proliferation and genomic instability in bladder cancer. Utilizing the GEPIA platform for validation purposes, it was determined that, except for MAPK1 and MAPK13, these genes exhibited significant expression levels in bladder cancer tissues (*p* < 0.01). Furthermore, an association between MAPK1 and MAPK3 with overall survival was determined. It should be noted that GEPIA-based survival analyses, due to their public and univariate nature, do not include clinical covariates; therefore, caution is required when interpreting the results. It was established that MAPK1 and MAPK3 represent therapeutically significant target components of the pathway [23,24,25]. It was demonstrated that hsa-miR-15a-5p, hsa-miR-16-5p, hsa-miR-195-5p, and hsa-miR-497-5p are strongly associated with cell cycle arrest, p53 activation, and apoptosis. This suggests that they have tumor-suppressing functions. Conversely, hsa-miR-19a-3p and hsa-miR-19b-3p exhibited enrichment in the MAPK, ErbB, and PI3K-Akt pathways, which are characteristic of aggressive tumor behavior. This finding indicates that these miRNAs may act as oncogenes. In light of these results, these molecules could be potential targets for future research into tumor progression and invasion in advanced bladder cancer [26,27]. Taken together, these findings suggest that only a few miRNA families are involved in key regulatory pathways that drive bladder cancer progression [28,29]. These findings provide a framework that may support future investigations into miRNome-based molecular profiling in bladder cancer, particularly in the context of hypothesis generation for diagnostic and prognostic research [30,31]. The members of the oncogenic miR-17~92 cluster, hsa-miR-17 and hsa-miR-20a, are each subject to regulation by six TFs; specifically, MYC, E2F1, STAT5, MYCN, NFKB1, and SPI1 for miR-17 and MYC, E2F1, STAT5, MYCN, ESR1, and SPI1 for miR-20a. These TFs are associated with cell cycle regulation, oncogenic signaling, and transcriptional control, thereby reinforcing the cluster’s central role in tumor progression [31]. The miR-200 family (including hsa-miR-200a, -200b, -200c, hsa-miR-141, and hsa-miR-429) is subject to regulation by five distinct TFs, namely ZEB1, TGFB1, TP53, SP1, and TWIST1. These TFs are pivotal to the processes of epithelial–mesenchymal transition (EMT) and tumor metastasis. This study demonstrated that TWIST1 is associated with MAPK1 and MAPK3 and is regulated by the miR-200 family. This suggests that TWIST1 may be involved in EMT programs associated with cell invasion and metastasis in bladder cancer [32,33,34]. These findings highlight the crucial role of TFs in controlling the biosynthesis, maturation, activation, and degradation of miRNAs within cells. The regulation of these processes is imperative for fundamental biological events such as cell fate, proliferation, and apoptosis, and their disruption is associated with numerous diseases, including cancer. These findings highlight the complex transcriptional control of cancer-associated miRNAs, suggesting that MYC, E2F1, and TP53 are key transcriptional regulators that play central roles in regulating miRNA-mediated gene regulatory networks in bladder cancer [35,36]. Hsa-miR-17-5p and hsa-miR-106a-5p target E2F1 (a cell cycle regulator) and are associated with ‘Pathways in Cancer’, ‘Bladder Cancer’ and ‘Cell Cycle’. Their effects may be significant through direct TF regulation and broader pathway modulation [37,38,39]. It is also worthy of note that has-miR-200a-3p has been strongly associated with ‘transcriptional dysregulation in cancer’ and bladder cancer in particular. This observation suggests the possibility of a role for has-miR-200a-3p in the abnormal gene expression that is characteristic of cancer [40,41]. In order to further refine the most significant miRNAs, it would be beneficial to ascertain whether any of the miRNAs targeting MYC or TWIST1 exhibit strong associations with bladder cancer or related pathways in a heatmap. However, it is notable that the hsa-miR-17/106 families (hsa-miR-17-5p and hsa-miR-106a-5p) and hsa-miR-200a-3p are of particular interest due to their combination of TF targeting and pathway relationships [41,42,43].

The present study is subject to the limitation that its findings are skewed towards phosphorylation and MAPK-related signaling pathways, as the nature of the platform utilized is designed to target kinase genes exclusively. Therefore, the results of this investigation must be interpreted as a specific reflection of our role in kinase signaling, rather than the entire genomic environment.

## 5. Conclusions

This comprehensive study highlights molecular regulatory patterns associated with bladder cancer progression and suggests candidate pathways and regulators for further investigation. Our study identified 200 DEGs, and functional analyses highlighted the predominance of phosphorylation, MAPK, and Ras signaling pathways, indicating significant disruptions in cellular communication. PPI analysis revealed key modules associated with intracellular signaling (e.g., the MAPK pathway) and cell cycle control (e.g., the p53 pathway). Twenty hub genes, including MAPK1, MAPK3, AKT1, and CDK1, were mainly validated and shown to play roles in cell cycle/signaling; MAPK1/3 was demonstrated to have prognostic significance. The regulatory network investigation revealed a complex interaction between TFs and miRNAs. Key TFs, including MYC, E2F1, and TP53, were identified as central regulators of both hub genes and miRNAs, suggesting transcriptional dysregulation. The identified PPI modules suggest coordinated dysregulation of kinase-driven signaling and cell cycle–related processes, highlighting groups of interacting proteins that may function within shared biological pathways rather than acting independently. The analysis of the DEGs queried through the TRRUST database revealed that a total of 6 TFs (Table 2), including E2F1 (5 genes), TP53 (4 genes), and E2F3 (3 genes), were enriched at an FDR significance threshold of 0.05. TFs such as E2F1 and TP53, which play central roles in cellular proliferation, cell cycle control, and apoptosis, were identified as the primary regulators of the DEGs in our study (FDR = 6.21 × 10^−7^ for E2F1). miRNAs, particularly tumor suppressors (e.g., miR-15a/16, miR-200 family), were associated with cell cycle arrest/apoptosis, while oncogenic ones (e.g., miR-19a/b-3p, miR-17~92 family) exerted their effects through aggressive tumor pathways (MAPK, PI3K-Akt). The integrated miRNA-TF-DEG regulatory network provided a holistic view of these multi-layered interactions, pinpointing critical nodes among TFs (MYC, TP53, E2F1), miRNAs (miR-17, miR-200, miR-29 families), and critical DEGs (MAPK3, AKT1, CHEK1). At the molecular level, this study reveals a coordinated dysregulation of kinase-driven signaling, cell cycle control, and transcriptional regulation in muscle-invasive bladder cancer. The integrated miRNA-TF-DEG network suggests that key transcription factors, including MYC, TP53, E2F family members, and TWIST1, centrally modulate aberrant MAPK signaling and cell cycle progression. miRNAs such as the miR-15/16, miR-17~92, and miR-200 families act as critical post-transcriptional regulators in this process. Together, these interactions point to a molecular framework in which proliferative signaling, genomic instability, and epithelial–mesenchymal transition are interconnected processes that contribute to MIBC progression. Although these findings are derived from in silico analyses and require experimental validation, they offer insight into the regulatory mechanisms underlying bladder cancer biology. The main limitation of the study is that the platform only includes 550 kinase genes. Consequently, the results reflect the analysis of a kinase-rich biological subset rather than the entire transcriptome. Furthermore, the findings are based solely on in silico analyses and require functional and clinical validation. Dependency on the patient cohort and the platform also limits generalizability.

## Figures and Tables

**Table 1:** Gene Expression Levels in Our Study and GEPIA Hub Genes.

Hub Gene	Regulation Effect in Our Study	GEPIA
MAPK1	Up Regulation	Down Regulation
AKT1	Down Regulation	Down Regulation
MAPK3	Down Regulation	Down Regulation
BUB1	Up Regulation	Up Regulation
CDK1	Up Regulation	Up Regulation
PLK1	Up Regulation	Up Regulation
MAPK14	Down Regulation	Down Regulation
ABL1	Down Regulation	Down Regulation
PRKACA	Down Regulation	Down Regulation
RAF1	Down Regulation	Down Regulation
AKT3	Down Regulation	Down Regulation
CHEK1	Up Regulation	Up Regulation
PRKACB	Down Regulation	Down Regulation
AURKA	Up Regulation	Up Regulation
AURKB	Up Regulation	Up Regulation
BUB1B	Up Regulation	Up Regulation
PRKCA	Down Regulation	Down Regulation
MAPK13	Down Regulation	Up Regulation
PBK	Up Regulation	Up Regulation

**Table 2:** TF related to hub gene.

Key TF	Description	# of Overlapped Genes	*p*-Value	Q Value	List of Overlapped Genes
E2F1	E2F transcription factor 1	5	1.80 × 10^−7^	7.38 × 10^−7^	CDK1, AURKB, CHEK1, AURKA, PLK1
E2F3	E2F transcription factor 3	3	2.46 × 10^−7^	7.38 × 10^−7^	PLK1, AURKA, CDK1
TP53	tumor protein p53	4	1.92 × 10^−5^	3.85 × 10^−5^	CHEK1, PLK1, AKT1, CDK1
E2F4	E2F transcription factor 4, p107/p130-binding	2	0.00024	0.00036	PLK1, AURKB
TWIST1	twist basic helix-loop-helix transcription factor 1	2	0.00056	0.000672	MAPK3, MAPK1
MYC	v-myc myelocytomatosis viral oncogene homolog (avian)	2	0.00448	0.00448	MAPK1, CHEK1

## Data Availability

This article does not involve data sharing as no new data were created or analyzed in the study. The raw data from the database were used. The work lines are listed below. The raw expression data from GSE130598 were obtained via the GEOquery R package (v2.66.0) (accession number: GSE130598, platform GPL26612). Enrichment analysis, GO, and KEGG were performed for DEGs (Using the ClusterProfiler (V 4.19.3) and enrichR packages). HUB gene analyses were conducted utilizing the STRING database. GEPIA was utilized to validate DEGs using the TCGA (The Cancer Genome Atlas) and GTEx (Genotype-Tissue Expression) datasets. The miRDB database was utilized for the analysis of miRNAs associated with DEG. In order to determine the key transcription factors (TFs) that regulate DEGs, the TRRUST v2 (Transcriptional Regulatory Relationships Unraveled by Sentence-based Text Mining) database was utilized. GSE130598 data: https://www.ncbi.nlm.nih.gov/geo/query/acc.cgi?acc=GSE130598 (accessed on 27 January 2025); GEOquery R paketi: https://bioconductor.org/ (accessed on 27 January 2025); KEGG: https://www.genome.jp/kegg/ (accessed on 27 January 2025); STRING: https://cn.string-db.org/ (accessed on 27 January 2025); GEPIA: http://gepia.cancer-pku.cn/ (accessed on 27 January 2025); MİRDB: https://mirdb.org/mining.html (accessed on 27 January 2025); TRRUST: https://ngdc.cncb.ac.cn/databasecommons/database/id/5213 (accessed on 27 January 2025).
